# The relationship between the radiation dose of pelvic-bone marrow and lymphocytic toxicity in concurrent chemoradiotherapy for cervical cancer

**DOI:** 10.1186/s13014-023-02205-8

**Published:** 2023-01-20

**Authors:** Bao-Zhong Zhang, Yang Li, Li-Ming Xu, Yan-Lan Chai, Chao Qu, Yuan-Jie Cao, Jing Wang, Hai-Ling Hou, Jiaqi Zhang

**Affiliations:** Department of Radiation Oncology, Tianjin Medical University Cancer Institute and Hospital, National Clinical Research Center for Cancer, Key Laboratory of Cancer Prevention and Therapy, Tianjin’s Clinical Research Center for Cancer, Tianjin, 300060 China

**Keywords:** Cervical cancer, Concurrent chemoradiotherapy, Intensity-modulated radiation therapy (IMRT), Lymphocytic toxicity, Dosimetry parameters

## Abstract

**Objective:**

The purpose of this study is to verify the correlation between medium and low radiation doses of the pelvic-bone marrow and the incidence of lymphocytic toxicity during concurrent chemoradiotherapy for cervical cancer.

**Materials and methods:**

This research included 117 cervical cancer patients, who received concurrent chemoradiotherapy. Radiotherapy included external-beam radiation therapy and brachytherapy. The dosimetry parameters include the Volume receiving 5 Gy (V5), 10 Gy (V10), 20 Gy (V20), 30 Gy (V30), 40 Gy (V40), 50 Gy (V50), and the mean dose (D mean) of the bone marrow. Lymphocytic toxicity was calculated from lowest lymphocytic count after two cycles of concurrent chemotherapy.

**Results:**

During concurrent chemoradiotherapy, the incidence of lymphocytic toxicity is 94.88%. The incidence of grade 3–4 toxicity is 68.38%. Multivariate analysis findings show that the dosimetry parameters V5, V10, V20, and V30 are significantly correlated with lymphocytic toxicity. The patients are divided into small-volume subgroups and large-volume subgroups based on the cutoff values. The relative risk of both grade 1–4 and grade 3–4 lymphocytic toxicity is significantly lower in the small-volume subgroups than in the large-volume subgroups (*P* < 0.05). Kaplan–Meier analysis shows that the incidence of both grade 1–4 and grade 3–4 lymphocytic toxicity of the small-volume subgroups is significantly lower than that of the large-volume subgroups (*P* < 0.05).

**Conclusion:**

There is a significant correlation between a medium and low dose of pelvic-bone-marrow radiation and incidence of lymphocytic toxicity. Reducing the volume of medium and low radiation doses could effectively reduce incidence of lymphocytic toxicity.

## Introduction

Cervical cancer is a common gynecological malignancy. It is the fourth most-common malignancy in females globally [1, 2]. Every year, there are 65,000 new cases of cervical cancer and 25,000 cervical cancer deaths in China. At present, concurrent chemoradiotherapy is the standard treatment for locally advanced cervical cancer [3, 4]. However, in the treatment process the hematologic toxicity clearly affects the intensity and progress of radiotherapy and chemotherapy, ultimately leading to poor outcomes in certain cases [5–7]. Clinical studies have confirmed that high doses and volumes of pelvic-bone-marrow irradiation can increase the risk of hematologic toxicities, and the skills of reducing dose and volume of bone-marrow are advised to lower the risk of hematologic adverse events [8, 9]. The use of intensity-modulated radiation therapy (IMRT) technology and Volumetric modulated arc therapy (VMAT) has efficaciously reduced the volume and dose of irradiation of the surrounding organs, but does not cause a decrease in the low-dose irradiation volume of normal tissue [10, 11]. In addition to neutrophil toxicity, erythrocyte toxicity, and platelet toxicity, lymphocytic toxicity is common during concurrent chemoradiotherapy of tumors [7–9, 12]. Peripheral lymphocytes play an important role in the treatment of tumors as a prominent component of the host’s immune system; lymphocytes act as safeguards and play a crucial role in the antitumor immune response by recognizing and destroying malignant cells [13–17]. The peripheral lymphocytes might either be killed directly by the X-ray during the radiotherapy, or be consumed during the tumor cell killing process. If the reduction of the lymphocytes is radiation dosage dependent, the lymphocytic toxicity should be taken into consideration during the radiotherapy. Therefore, in this study, we retrospectively analyzed 117 cervical cancer patients, all of whom had received concurrent chemoradiotherapy, to prove the correlation between the volume of medium- and low-dose radiation of the pelvic-bone marrow and lymphocytic toxicity during treatment. We hope that this study can provide a basis for optimizing an IMRT/ VMAT plan and reduce the volume of medium- and low-dose radiation of bone marrow for the aim to reduce the incidence of lymphocytic toxicity.

## Material and methods

### Patients

We retrospectively analyzed the patients who received concurrent chemoradiotherapy between January 2016 and December 2018 in our hospital; all these patients were pathologically diagnosed with cervical squamous carcinoma or adenocarcinoma. The inclusion criteria were as follows: (1) the cancer was staged IIA-IIIC (based on the 2014 International Federation of Gynecology and Obstetrics’ (FIGO) staging, carried out by gynecological examination and imaging examination (CT or MRI)), for stage I, the patients received surgical treatment, for stage IV the patients received chemotherapy as the main treatment; (2) patients were between the ages of 18 and 70 years; (3) patients’ Karnofsky performance status (KPS)scores were ≥ 70; (4) complete records of blood were available from routine examinations at the time of pretreatment, and were taken weekly during the treatment and within a month of the treatment’s end. The exclusion criteria were: (1) that the concurrent chemotherapy finished in fewer than three cycles; (2) that the interruption interval of radiotherapy was longer than one week for any reason; (3) that patients had a second primary tumor; (4) that patients had a history of radiotherapy. With these criteria, a total of 117 patients were included in the study.

### Radiotherapy

All 117 patients received external-beam radiation therapy (EBRT) and brachytherapy as treatment, as detailed below.

EBRT: The 6MV-X ray-linear accelerator was used. The radiation plan was designed on the images of simulate CT scan with the patients prone. The clinical target volume (CTV) was defined as the gross tumor, the cervix, the entire uterus, the vagina, he obturator lymph node drainage area, and the internal and external iliac lymph node drainage area, the inferior border at 3 cm below the most inferior vaginal involvement, and the superior border was at the lumbar 4/5 level. All of the diagnosed metastatic lymph nodes in the pelvic cavity were defined as the gross tumor target volume of lymph node (GTVnd). The planning target volume (PTV) of the CTV was to add 0.7 cm laterally and 1.5 cm axially on the primary CTV; the PTV of the GTVnd (PGTVnd) was to add 0.7 cm laterally and 1.5 cm axially on primary GTVnd. The total dose delivered to the PGTVnd was 59.92 Gy (2.14 Gy per fraction, with 28 fractions). The total dose delivered to the PTV was 50.4 Gy (1.8 Gy per fraction, with 28 fractions). The radiation therapy progressed as one fraction per day, five days per week. The contouring of the organ at risk included the small intestine, rectum, bladder, and the femoral head.

Brachytherapy: brachytherapy was performed using Iridium-192. The dose tare was 12–70 Gy/h. Brachytherapy started within one week of the EBRT, and the total dose was 28 Gy (7 Gy per fraction per week, for four weeks).

### Induction chemotherapy

Patients with a FIGO stage of between IIIA and IIIB were advised to receive one cycle of induction chemotherapy before concurrent chemoradiotherapy. The regimen consisted of paclitaxel (175 mg/m^2^) and cisplatin (75 mg/m^2^). Concurrent chemoradiotherapy started three weeks after induction chemotherapy.

### Concurrent chemotherapy

The concurrent chemotherapy regimen was a weekly dose of cisplatin (25 mg/m^2^), starting at the first week of EBRT and with a total of five cycles planned. Chemotherapy was stopped when the white blood cell (WBC) count was lower than 2.0*10^9^/L or the neutrophil count was lower than 1.5*10^9^/L.

### Dosimetry parameters of the bone marrow

The contouring of the bone marrow included the lower lumbar spine (the superior border depending on the superior border of the PTV), the sacrum, the ilium, and the upper femur (the inferior border depending on the inferior border of the PTV); the bone cortex was also included. The volume dosimetry parameter was defined as the volume(ml) of a definite radiation dose, such as V5 means the total volume of the bone that received the radiation dose of 5 Gy. And the dosimetry parameters included the V5, V10, V20, V30, V40, V50, and the mean dose of the bone marrow (Dmean).

### The lymphocytic count

The lymphocyte was counted in routine blood tests before, during, and after the concurrent chemoradiotherapy. Final lymphocytic toxicity was defined as the lowest lymphocytic count after two cycles of concurrent chemotherapy, either during the chemoradiotherapy or within one month of it. Lymphocytic toxicity was defined from grade 0 to 4, based on the common terminology criteria for adverse events version 4.0: grade 0 (normal) is a value ≥ 0.80 × 10^9^/L; grade 1 is a value 0.60–0.79 × 10^9^/L; grade 2 is a value 0.40–0.59 × 10^9^/L; grade 3 is a value 0.20–0.39 × 10^9^/L; and grade 4 is a value < 0.20 × 10^9^/L.

### Statistical analysis

The correlation between the clinicopathologic features and the lymphocytic toxicity was analyzed using multivariate analysis and univariate regression analysis model. The correlation between the dosimetry parameters and the lowest lymphocytic count was analyzed by linear regression. The cutoff values of the dosimetry parameters were obtained using receiver operating characteristic (ROC) curves. The patients were divided into subgroups based on the cutoff values of the dosimetry parameters. The relative risk (RR) was analyzed with χ2 tests. A comparison of the incidence of lymphocytic toxicity between the dosimetry subgroups was analyzed using the Kaplan–Meier method. Data were assessed with IBM-SPSS software version 22.0, and all values of p < 0.05 were considered statistically significant.

## Results

### The clinicopathologic features

There were 117 patients included in this study, with a median age of 54 (29–70) years. The total lymphocytic toxicity (grade 1–4) rate was 94.88%, and the grade 3–4 lymphocytic toxicity rate being 68.38%. Table 1 shows these results.

**Table 1:** The clinicopathologic features

Index	n (%)
Age	
< 60	83 (70.94)
≥ 60	34 (29.06)
Pathology	
Squamous cell carcinoma	97 (81.91)
Adenocarcinoma	20 (17.09)
FIGO stage	
IIA	26 (22.22)
IIB	62 (52.99)
IIIA	8 (6.84)
IIIB	16 (13.68)
IIIC	5 (4.27)
Radiation technique	
IMRT	29 (24.79)
VMAT	88 (75.21)
KPS score	
80–90	57 (48.72)
> 90	60 (51.28)
Induction chemotherapy	
Yes	92 (78.63)
No	25 (21.37)
Concurrent chemotherapy circles	
3–4 circles	66 (56.41)
5 circles	51 (43.59)
Lymphocytotoxicity	
0	6 (5.12)
1–4	111 (94.88)
3–4	80 (68.38)

*FIGO* Federation International Of Gynecology And Obstetrics, *IMRT* intensity-modulated radiation therapy, *VMAT* volume-modulated arc therapy, *KPS* Karnofsky Performance Status

### Multivariate analysis of the dosimetry parameters for the lymphocytic toxicity

We performed multivariate analysis that considered the clinicopathologic features of age, pathology, FIGO stage, KPS score, induction chemotherapy, and concurrent chemotherapy cycles, together with dosimetry parameters. the endpoint was the occurrence of grade 1 lymphocytic toxicity. The results, shown in Table 2, demonstrate that V5 (*P* = 0.004), V10 (*P* = 0.006), V20 (*P* = 0.020), and V30 (*P* = 0.016) were correlated with the lymphocytic toxicity. But the age, pathology, FIGO stage (on behalf of the size of the tumor), introduction chemotherapy, concurrent chemotherapy cycles and KPS score were not correlated with the lymphocytic toxicity (data not shown).

**Table 2:** The multivariate analysis of V5, V10, V15, V20, V30 for the lymphocytic toxicity

	OR	95% CI	*P*
V5			
Group 1 (< 1015.775 ml)	1		
Group 2 (≥ 1015.775 ml)	1.513	1.106–1.819	0.004
V10			
Group 1 (< 950.15 ml)	1		
Group 2 (≥ 950.15 ml)	1.415	1.024–1.766	0.006
V20			
Group 1 (< 810.22 ml)	1		
Group 2 (≥ 810.22 ml)	1.536	1.110–1.898	0.020
V30			
Group 1 (< 792.205 ml)	1		
Group 2 (≥ 792.205 ml)	1.557	1.124–1.817	0.016

### Correlation between the dosimetry parameters and the lowest lymphocytic count, and the ROC curves

**Fig. 1:**
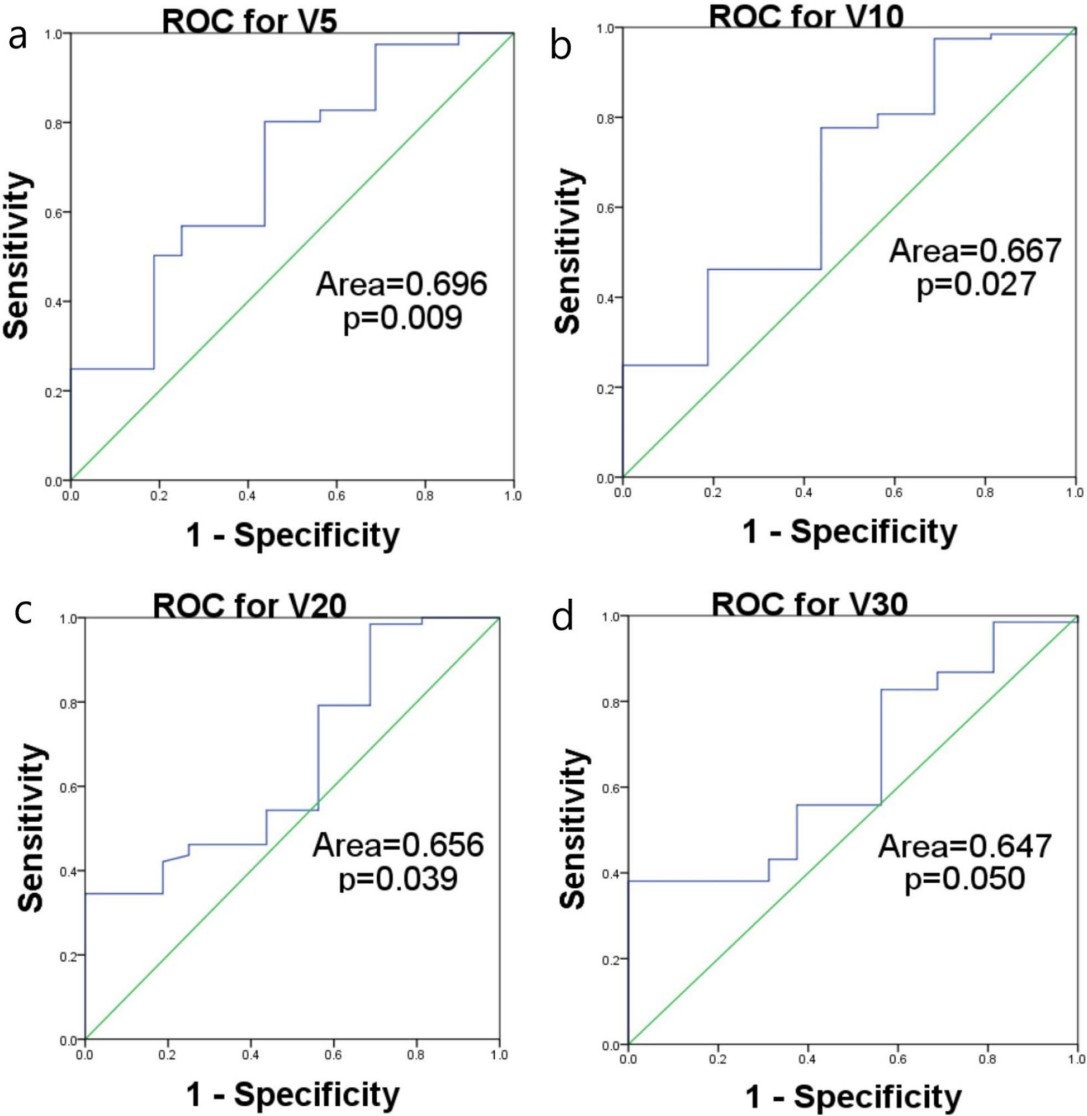
ROC curves for the dosimetry parameters. **a** ROC for V5 (area = 0.696, *P* = 0.009); **b** ROC for V10 (area = 0.667, *P* = 0.027); **c** ROC for V20 (area = 0.656, *P* = 0.039); **d** ROC for V30 (area = 0.647, *P* = 0.050)

The dosimetry parameters of V5, V10, V20, and V30 were found to correlate with the lowest lymphocytic count, while the dosimetry parameters of V40, V50, and Dmean were not correlated. These results are shown in Table 3. ROC curves were then made for the dosimetry parameters of V5, V10, V20, and V30, the endpoint was the occurrence of grade 1 lymphocytic toxicity. The cutoff values were obtained, and the patients were divided into small-volume (group 1) and large-volume (group 2) subgroups, based on the cutoff values of the V5, V10, V20, and V30. For V5, a volume < 1015.78 ml went into group 1, and a volume ≥ 1015.78 ml went into group 2; for V10, a volume < 950.15 ml went into group 1, and a volume ≥ 950.15 ml went into group 2; for V20, a volume < 810.22 ml went into group 1, and a volume ≥ 810.22 ml went into group 2; for V30, a volume < 792.21 ml went into group 1, and a volume ≥ 792.21 ml went into group 2. See Table 4 for these results. And the ROC curves were showed in Fig. 1.

**Table 3:** The results of linear regression between the dosimetry parameters and the lowest lymphocytic count

	F	Adjusted R^2^ (SD)	*P*
V5	25.206	0.273 (0.171)	< 0.001
V10	24.894	0.271 (0.133)	< 0.001
V20	22.179	0.266 (0.172)	< 0.001
V30	11.345	0.252 (0.180)	0.001
V40	4.717	0.131 (0.185)	0.062
V50	1.855	0.126 (0.187)	0.176
Dmean	0.834	0.085 (0.088)	0.363

**Table 4:** The results the ROC curves and the cutoff values

	ROC area (SD)	*P*	Cutoff value (ml)
V5	0.696 (0.070)	0.009	1015.78
V10	0.667 (0.072)	0.027	950.15
V20	0.656 (0.068)	0.039	810.22
V30	0.647 (0.064)	0.050	792.21

### The χ^2^ test for the RR of lymphocytic toxicity between the subgroups of every dosimetry parameter

For grade 1–4 lymphocytic toxicity, the RR of group 1 and group 2 for V5, V10, V20, and V30 were 0.793, 0.818, 0.915, and 0.922, respectively. For grade 3–4 lymphocytic toxicity, the RR of group 1 and group 2 for V5, V10, V20, and V30 were 0.700, 0.587, 0.673, and 0.668, respectively. These results are shown in Table 5.

**Table 5:** The results of the RR for grade 1–4 and grade 3–4 lymphocytic toxicity between subgroups

	RR (Group 1 vs. Group 2)	95% CI
Grade 1–4		
V5	0.793	0.659–0.955
V10	0.818	0.697–0.961
V20	0.915	0.813–1.029
V30	0.922	0.864–0.984
Grade 3–4		
V5	0.700	0.482–1.017
V10	0.587	0.397–0.869
V20	0.673	0.481–0.943
V30	0.668	0.535–0.834

### Kaplan–Meier analysis for lymphocytic toxicity between subgroups of every dosimetry parameter

**Fig. 2:**
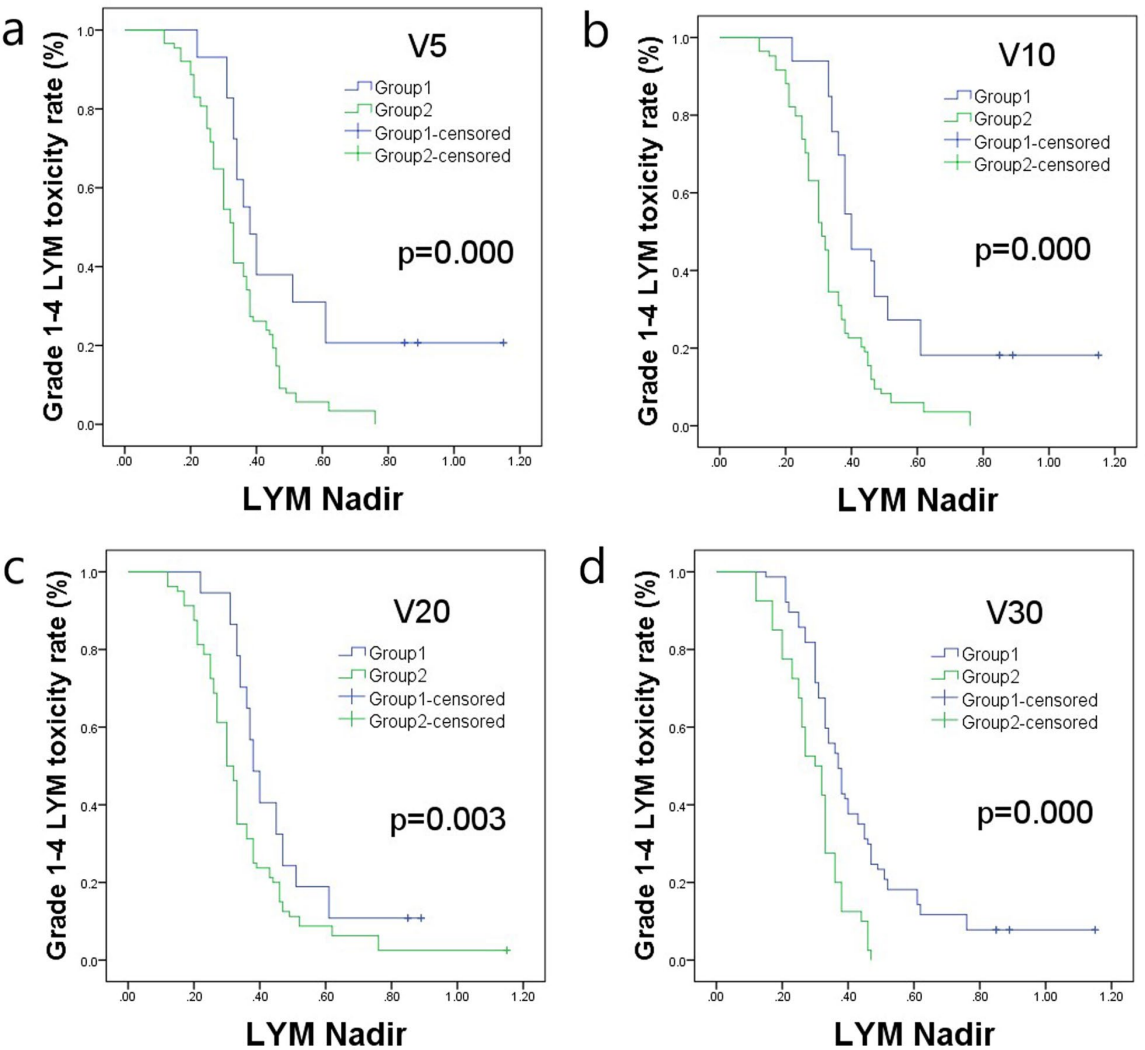
Kaplan–Meier analysis of grade 1–4 lymphocytic toxicity for the dosimetry parameters. **a** For V5, *P* = 0.000; **b** for V10, *P* = 0.000; **c** for V20, *P* = 0.003; **d** for V30, *P* = 0.000

**Fig. 3:**
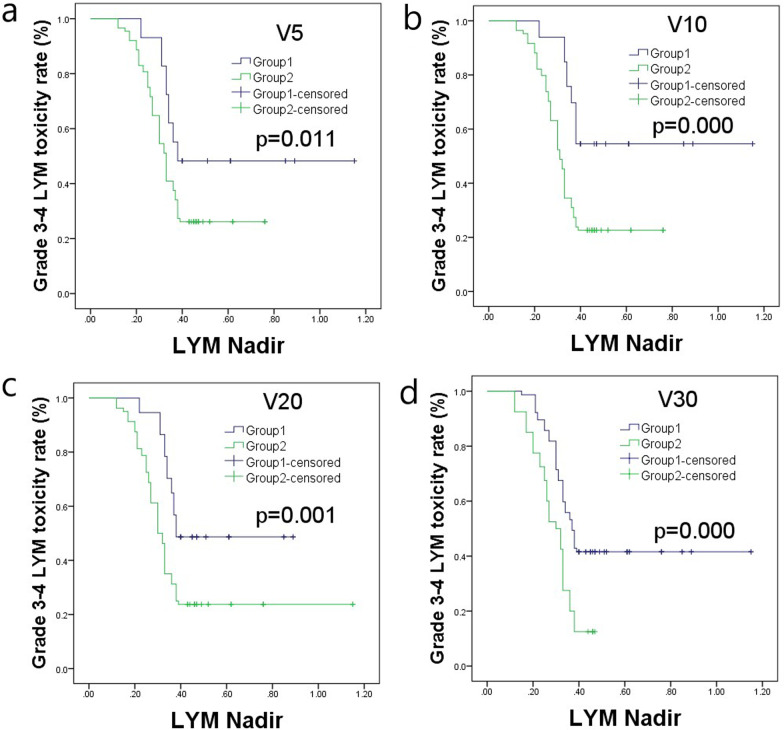
Kaplan–Meier analysis of grade 3–4 lymphocytic toxicity for the dosimetry parameters. **a** For V5, *P* = 0.011; **b** for V10, *P* = 0.000; **c** for V20, *P* = 0.001; **d** for V30, *P* = 0.000

The Kaplan–Meier analysis showed that, for grade 1–4 lymphocytic toxicity, there was a significant difference between the subgroups of V5, V10, V20, and V30, and the *P*-values were 0.000, 0.000, 0.003, and 0.000, respectively. For grade 3–4 lymphocytic toxicity, there were significant differences between the subgroups of V5, V10, V20, and V30, and the *P*-values were 0.011, 0.000, 0.001, and 0.000, respectively. The results were showed in Figs. 2 and 3, the data was showed in Table 6.

**Table 6:** The K-M analysis for grade 1–4 and grade 3–4 lymphocytic toxicity between subgroups of every dosimetry parameter (median ± SD)

	The lowest lymphocyte count of Group 1	The lowest lymphocyte count of Group 2	*P*
Grade 1–4			
V5	0.507 ± 0.049	0.343 ± 0.014	0.000
V10	0.508 ± 0.042	0.334 ± 0.015	0.000
V20	0.450 ± 0.029	0.353 ± 0.021	0.003
V30	0.430 ± 0.024	0.294 ± 0.015	0.000
Grade 3–4			
V5	0.380 ± 0.008	0.330 ± 0.013	0.011
V10	0.400 ± 0.010	0.280 ± 0.011	0.000
V20	0.380 ± 0.008	0.200 ± 0.013	0.001
V30	0.370 ± 0.018	0.200 ± 0.027	0.000

## Discussion

Hematological adverse event is common in radiotherapy of pelvic tumors, especially in concurrent chemoradiotherapy. When serious, it limits the intensity of radiotherapy and chemotherapy, prolongs the duration of treatment, and ultimately leads to the treatment having limited impact [6, 7, 12, 18, 19]. Mell et al. have confirmed that a high dose and volume of pelvic-bone-marrow irradiation will increase the incidence of hematological toxicities. Use of IMRT technology and VMAT has effectively reduced the volume and dose of irradiation of the normal tissue, but the low-dose irradiation volume, which might also cause hematologic adverse events, does not decrease under IMRT technology and VMAT [10, 11]. It is reported the quite a large part of a patient’s total active bone marrow (nearly 50%) is within the pelvis and lumbar spine [20]. Therefore, during the radiation of the pelvic tumor, the protection of the bone marrow of the pelvis and lumbar spine seems very important.

The bone-marrow exposure is proved to relate to the hematological toxicity during pelvic radiation, and reducing the radiation dose of bone marrow could reduce the risk of clinical hematological toxicity [8, 21]. The published results found that bone-marrow-sparing IMRT could reduce acute hematology for patients with locally advanced cervical cancer; the dosimetry parameters used were the mean dose, V10, and V20 [22, 23]. Most published results were focused on the neutrophil toxicity, erythrocyte toxicity, and platelet toxicity, and the clinical treatments are effective for these adverse events. However, lymphocytic toxicity is often overlooked, and when the peripheral lymphocytes reduce, there are few effective treatments. Peripheral lymphocytes play an important role in the treatment of tumors as a prominent component of the host’s immune system; lymphocytes act as safeguards and play a crucial role in the antitumor immune response by recognizing and destroying malignant cells [13, 17]. A high pre-operative lymphocytic count has been proved to be independently correlated with a better prognosis for resectable cervical cancer, pancreatic cancer, breast cancer, and non-small-cell lung cancer [24–27]. The mechanisms by which lymphocytic count influences the outcomes can be explained by the functions of the subpopulations of lymphocytes. For example, the function of CD8 + and CD4 + T cells and the cytokines secreted by them might mediate the apoptosis of malignant cells and the antitumor response [28, 29]. From this it is clear that the prevention of lymphocytic toxicity is important.

Based on these theories, we aimed to find the correlation between the dosimetry parameters of the pelvic-bone marrow and the lymphocytic toxicity in concurrent chemoradiotherapy of the locally advanced cervical cancer, and, meanwhile, to prove the importance of adjusting the radiotherapy plan to reduce the risk of lymphocytic toxicity. As the results show, the volume of medium- and low-dose radiation significantly correlated with the lymphocytic toxicity. This is somewhat different from the cases of neutrophil toxicity, perhaps because the peripheral lymphocytes are more sensitive to radiation doses than the neutrophils. Another possible reason is that lymphocytes are consumed in the antitumor response. Subgroup analysis of the dosimetry parameters V5, V10, V20, and V30 show that the incidence of both grade 1–4 and grade 3–4 lymphocytic toxicity of the small-volume subgroups was lower than that of the large-volume subgroups. Taking the consumption of antitumor response into consideration, the reduction of the volume of medium- and low- dose radiation of pelvic-bone marrow might be beneficial for the treatment of cancer. Most previous studies have reported the hematologic toxicity, specifically neutropenia and its impact. Our study focuses instead on lymphocytic toxicity associated with bone-marrow dose, an aspect usually overlooked by clinicians and researchers. The results remind us that it is necessary to adjust radiotherapy plans for the purpose of maintaining an adequate lymphocyte count through bone marrow sparing.

There are several limitations in our study. This is a retrospective piece of research, so our results should be confirmed by further prospective studies. The mesenteric lymph node is another aggregation area of lymphocytic rank, second only to bone marrow; though the radiation dose of the mesenteric lymph nodes was low, it might influence the peripheral lymphocytic count, but this was not taken into consideration in this research.

## Conclusion

During the process of concurrent chemoradiotherapy for cervical cancer, there is a significant correlation between the volume of medium (V20, V30)—and low(V5, V10)-dose radiation of bone marrow within the radiotherapy range and the incidence of lymphocytic toxicity. Reducing the volume of medium- and low-dose radiation could effectively reduce the incidence of lymphocytic toxicity.

## Data Availability

The datasets used and analyzed during the current study are available from the corresponding author on reasonable request.

## Abbreviations

IMRT: Intensity-modulated radiation therapy
VMAT: Volumetric modulated arc therapy
EBRT: External-beam radiation therapy
FIGO: International Federation of Gynecology and Obstetrics
KPS: Karnofsky performance status
CTV: Clinical target volume
GTVnd: Gross tumor target volume of lymph node
PTV: Planning target volume
WBC: White blood cell
ROC: Receiver operating characteristic
RR: The relative risk
